# Androgen receptor activity modulates responses to cisplatin treatment in bladder cancer

**DOI:** 10.18632/oncotarget.9994

**Published:** 2016-06-14

**Authors:** Eiji Kashiwagi, Hiroki Ide, Satoshi Inoue, Takashi Kawahara, Yichun Zheng, Leonardo O. Reis, Alexander S. Baras, Hiroshi Miyamoto

**Affiliations:** ^1^ Departments of Pathology and Urology, Johns Hopkins University School of Medicine, Baltimore, MD, USA

**Keywords:** androgen receptor, bladder cancer, chemoresistance, cisplatin, NF-κB

## Abstract

Cisplatin (CDDP)-based combination chemotherapy remains the mainstream treatment for advanced bladder cancer. However, its efficacy is often limited due to the development of resistance for which underlying mechanisms are poorly understood. Meanwhile, emerging evidence has indicated the involvement of androgen-mediated androgen receptor (AR) signals in bladder cancer progression. In this study, we aimed to investigate whether AR signals have an impact on sensitivity to CDDP in bladder cancer cells. UMUC3-control-short hairpin RNA (shRNA) cells with endogenous AR and AR-negative 647V/5637 cells stably expressing AR were significantly more resistant to CDDP treatment at its pharmacological concentrations, compared with UMUC3-AR-shRNA and 647V-vector/5637-vector control cells, respectively. A synthetic androgen R1881 significantly reduced CDDP sensitivity in UMUC3, 647V-AR, or 5637-AR cells, and the addition of an anti-androgen hydroxyflutamide inhibited the effect of R1881. In these AR-positive cells, R1881 treatment also induced the expression levels of NF-κB, which is known to involve CDDP resistance, and its phosphorylated form, as well as nuclear translocation of NF-κB. In CDDP-resistant bladder cancer sublines established following long-term culture with CDDP, the expression levels of AR as well as NF-κB and phospho-NF-κB were considerably elevated, compared with respective control sublines. In bladder cancer specimens, there was a strong trend to correlate between AR positivity and chemoresistance. These results suggest that AR activation correlates with CDDP resistance presumably via modulating NF-κB activity in bladder cancer cells. Targeting AR during chemotherapy may thus be a useful strategy to overcome CDDP resistance in patients with AR-positive bladder cancer.

## INTRODUCTION

The standard of non-surgical care for bladder cancer following transurethral tumor resection includes systemic chemotherapy. Clinical studies have indeed demonstrated a survival benefit of neoadjuvant or adjuvant chemotherapy for patients with invasive bladder cancer before or after radical cystectomy [1, 2]. Cisplatin [*cis*-diamminedichloroplatinum(II); CDDP]-based chemotherapy (*e.g.* “MVAC”, “GC”) also constitutes the major therapeutic option for metastatic urothelial cancer [3]. However, a significant amount of patients with bladder cancer fail to have successful responses to systemic chemotherapy. Moreover, other patients who initially respond to CDDP therapy often acquire resistance in the end. Thus, the prediction of chemosensitivity as well as the development of chemosensitization strategies constitutes a goal with critical clinical implications.

Men are approximately three times more likely to develop bladder cancer than women [4]. Emerging preclinical evidence has suggested a critical role of androgen receptor (AR) signaling in inducing urothelial carcinogenesis and cancer progression [5–18], which may explain the gender-specific difference in the incidence of bladder cancer. Retrospective cohort studies have also revealed that androgen deprivation therapy (ADT) which has been widely used for the treatment of prostate cancer prevents the development of *de novo* [19] and recurrent [20] bladder tumors in male patients. Immunohistochemical studies in bladder cancer specimens have further indicated correlations between AR expression and disease progression, while no significant difference in its levels between male and female tumors has been found [21, 22]. Of note, androgen was recently shown to reduce sensitivity of AR-positive bladder cancer cells to doxorubicin [23], an anthracycline anti-tumor antibiotic often used for intravesical chemotherapy to prevent tumor recurrence. In addition, we have recently demonstrated, in bladder cancer cells, that androgens up-regulate ELK1 [15], a transcription factor whose downstream target is *c-fos* proto-oncogene, and that ELK1 inactivation results in enhancement of the cytotoxic activity of CDDP [24].

Based on these previous observations, we anticipated that AR activity could correlate with chemosensitivity. In the current study, we therefore assessed whether AR activation induced resistance to CDDP treatment in bladder cancer cells.

## RESULTS

### AR activation correlates with resistance to CDDP

**Figure 1:**
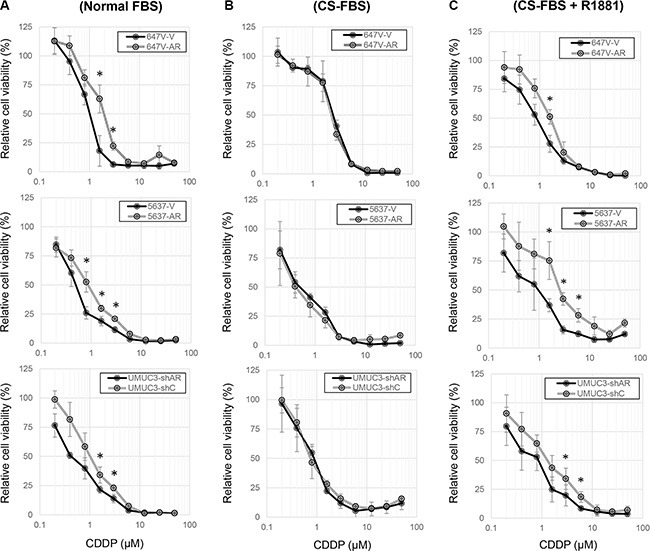
Effects of AR expression on the cytotoxicity of CDDP in bladder cancer cells MTT assay was performed in 647V-V/AR, 5637-V/AR, and UMUC3-control-shRNA (shC)/AR-shRNA (shAR) cells cultured in medium containing (**A**) 10% normal FBS, (**B**) 10% CS-FBS, or (**C**) 10% CS-FBS + 10 nM R1881, as well as different concentrations (0.2–50 μM) of CDDP for 72 h. Cell viability is presented relative to that of each line without CDDP treatment. Each value represents the mean (± SD) from at least three independent experiments. *P < 0.05 (647V-V *vs.* 647V-AR, 5637-V *vs.* 5637-AR, or UMUC3-control-shRNA *vs.* UMUC3-AR-shRNA).

MTT assay was used for assessing the viability of cells cultured in the presence or absence of CDDP and androgens. We first compared the cytotoxic effects of CDDP between AR-positive and AR-negative bladder cancer cell lines. CDDP inhibited cell growth in a dose-dependent manner, and AR-positive cells (*i.e.* 647V-AR and 5637-AR with exogenous AR, UMUC3-control-shRNA with endogenous AR) were more resistant to CDDP treatment at its pharmacological concentrations (*e.g.* 1.3 – 8.4 μM [25]), compared with respective AR-negative control lines (Figure 1A). In contrast, there were no significant differences in the effects of CDDP in AR-positive versus AR-negative cells when cultured in medium supplemented with charcoal-stripped fetal bovine serum (CS-FBS) (Figure 1B). In this androgen-depleted condition, however, addition of a synthetic non-metabolized androgen R1881 in medium resulted in considerable decreases in CDDP sensitivity in AR-positive cells (Figure 1C). In these assays, the effects of AR expression and/or androgen treatment on cell viability, irrespective of CDDP, were excluded by comparing with respective controls without CDDP treatment. These results suggest that AR activation reduces the cytotoxic activity of CDDP in bladder cancer cells.

**Figure 2:**
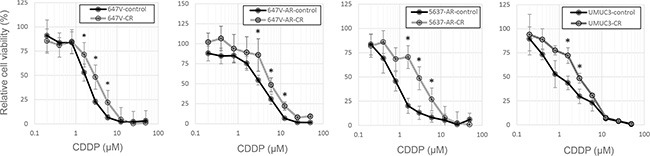
Establishment of CR sublines MTT assay was performed in 647V-control/CR, 647V-AR-control/CR, 5637-AR-control/CR, and UMUC3-control/CR cells cultured in medium containing 10% FBS as well as different concentrations (0.2–50 μM) of CDDP for 72 h. Cell viability is presented relative to that of each line without CDDP treatment. Each value represents the mean (± SD) from at least three independent experiments. *P < 0.05 (647V-control *vs.* 647V-CR, 647V-AR-control *vs.* 647V-AR-CR, 5637-AR-control *vs.* 5637-AR-CR, or UMUC3-control *vs.* UMUC3-CR).

**Figure 3:**
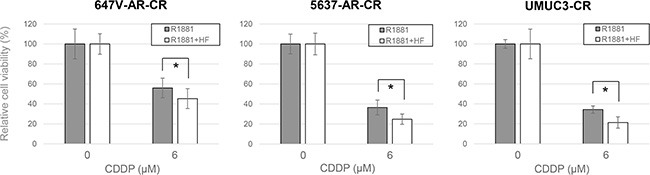
Effects of anti-androgen on the cytotoxicity of CDDP in CR bladder cancer cells cultured with androgen MTT assay was performed in 647V-AR-CR, 5637-AR-CR, and UMUC3-CR cells cultured in medium containing 10% CS-FBS, 10 nM R1881, either ethanol or 5 μM HF, and either ethanol or 6 μM CDDP for 72 h. Cell viability is presented relative to that of each line without CDDP treatment. Each value represents the mean (± SD) from at least three independent experiments. *P < 0.05 (R1881 *vs.* R1881 + HF in each cell line).

To further investigate the involvement of AR signals in CDDP resistance in bladder cancer cells, we established “CDDP-resistant (CR)” sublines by long-term culture with low/increasing doses of CDDP. The growth rates of the CR sublines were similar to those of control lines cultured for the same period without CDDP (data not shown). We then confirmed lower sensitivity to CDDP in CR sublines, compared with their controls (Figure 2). Nonetheless, in these CR sublines, treatment with an anti-androgen hydroxyflutamide (HF) increased CDDP sensitivity (Figure 3). Again, in this assay, the effects of androgen/anti-androgen treatment on cell viability, irrespective of CDDP, were excluded by comparing with respective controls without CDDP treatment.

### AR signaling correlates with nuclear factor (NF)-κB activity

**Figure 4:**
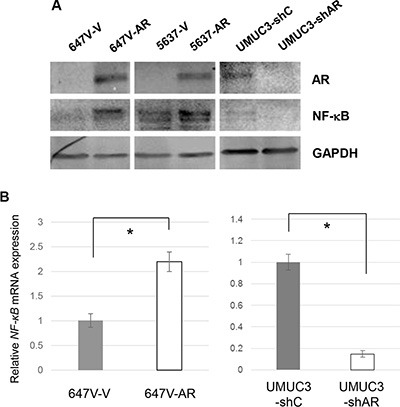
Effects of AR on NF-κB expression in bladder cancer cells (**A**) Western blotting of AR and NF-κB in 647V-V/AR, 5637-V/AR, and UMUC3-control-shRNA (shC)/AR-shRNA (shAR) cells. Total protein extracted from each line was immunoblotted for AR (110 kDa), NF-κB (65 kDa). GAPDH (37 kDa) served as an internal control. (**B**) Quantitative RT-PCR of *NF-κB* in 647V-V/AR and UMUC3-control-shRNA (shC)/AR-shRNA (shAR) cells. Each line was subjected to RNA extraction and subsequent real-time RT-PCR. Expression of *NF-κB* gene was normalized to that of *GAPDH*. Transcription amount is presented relative to that of each control line. Each value represents the mean (+SD) from at least three independent experiments. *P < 0.05 (647V-V *vs.* 647V-AR or UMUC3-control-shRNA *vs.* UMUC3-AR-shRNA).

**Figure 5:**
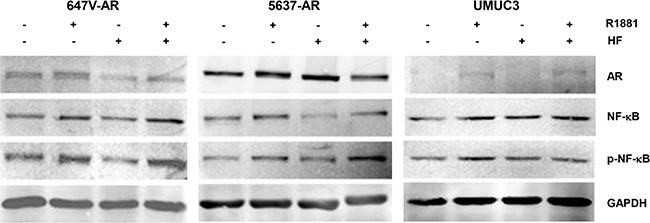
Effects of androgen on NF-κB expression in bladder cancer cells Western blotting of AR, NF-κB, and p-NF-κB in 647V-AR, 5637-AR, and UMUC3 cells. Total protein extracted from each line treated with ethanol (mock), 10 nM R1881, and/or 5 μM HF for 48 hours was immunoblotted for AR (110 kDa), NF-κB (65 kDa), or p-NF-κB (65 kDa). GAPDH (37 kDa) served as an internal control.

**Figure 6:**
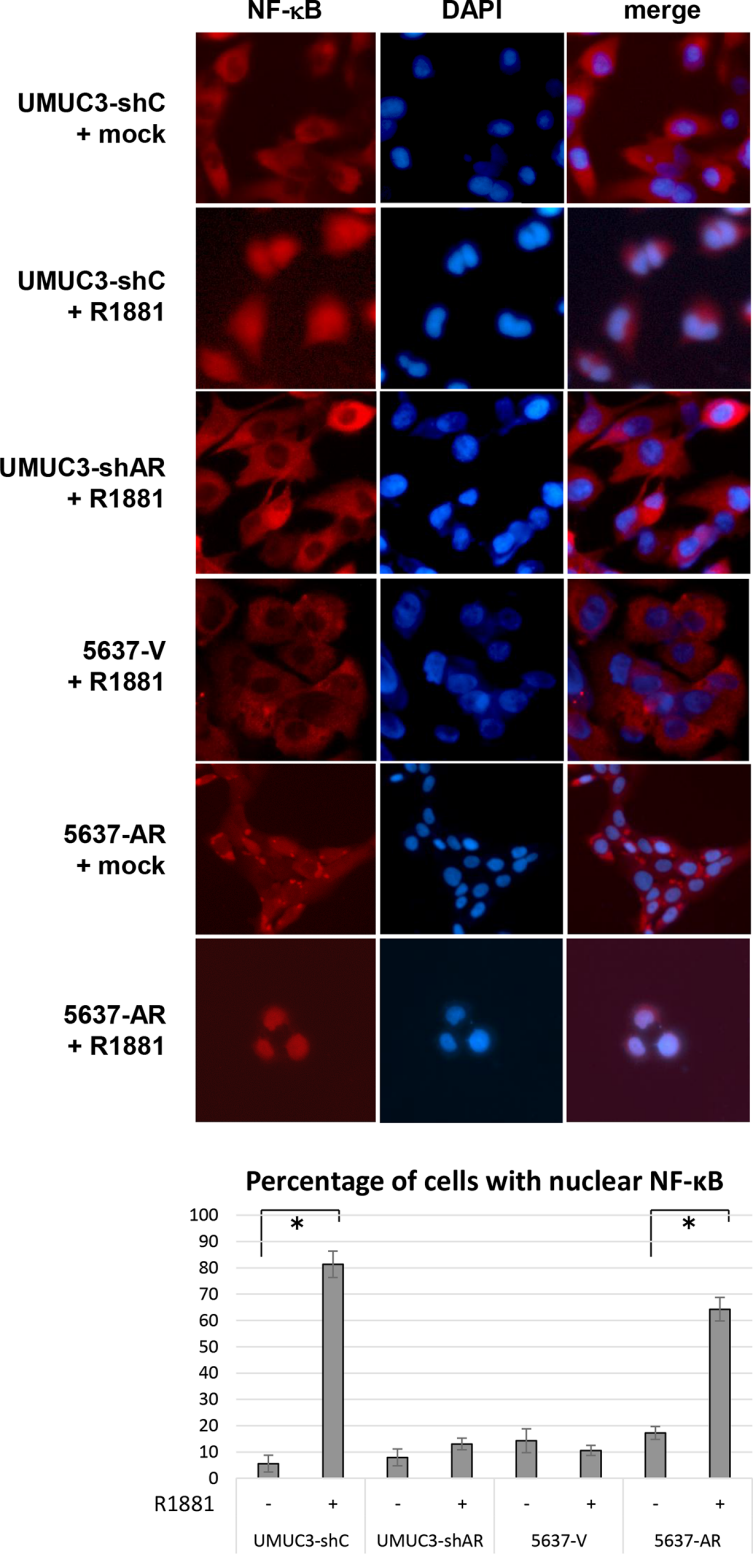
Effects of androgen on nuclear translocation of NF-κB in bladder cancer cells UMUC3-control-shRNA (shC)/AR-shRNA (shAR) and 5637-V/AR cells treated with ethanol (mock) or 10 nM R1881 for 24 h were analyzed on immunofluorescence, using an antibody to NF-κB. DAPI was used to visualize nuclei. The number of nuclear staining per visual field was quantified in five randomly selected visual fields per chamber. Each value represents the mean (+ SD) from at least three independent experiments. *P < 0.05 (mock *vs.* R1881).

NF-κB activation has been implicated in acquisition of CDDP resistance [26, 27]. We therefore investigated whether androgens/AR regulate the expression and activity of NF-κB in bladder cancer cells. Western blot and real-time reverse transcription (RT)-polymerase chain reaction (PCR) showed that AR-positive lines expressed higher levels of NF-κB protein (Figure 4A) and mRNA (Figure 4B), respectively, compared with AR-negative control lines. Similarly, in AR-positive cells, R1881 increased the expression of AR as well as that of NF-κB and its active form, phospho-NF-κB (p-NF-κB), and HF at least partially abolished the effect of R1881 on their expression (Figure 5). More remarkably, immunofluorescence that was performed to determine the localization of NF-κB showed its distribution predominantly in the cytoplasm of mock-treated cells and induction of its nuclear translocation by R1881 treatment in AR-positive cells, but not in AR-negative cells (Figure 6). Thus, androgen appeared to activate NF-κB via the AR pathway in bladder cancer cells.

### AR and NF-κB are up-regulated in CR cells

**Figure 7:**
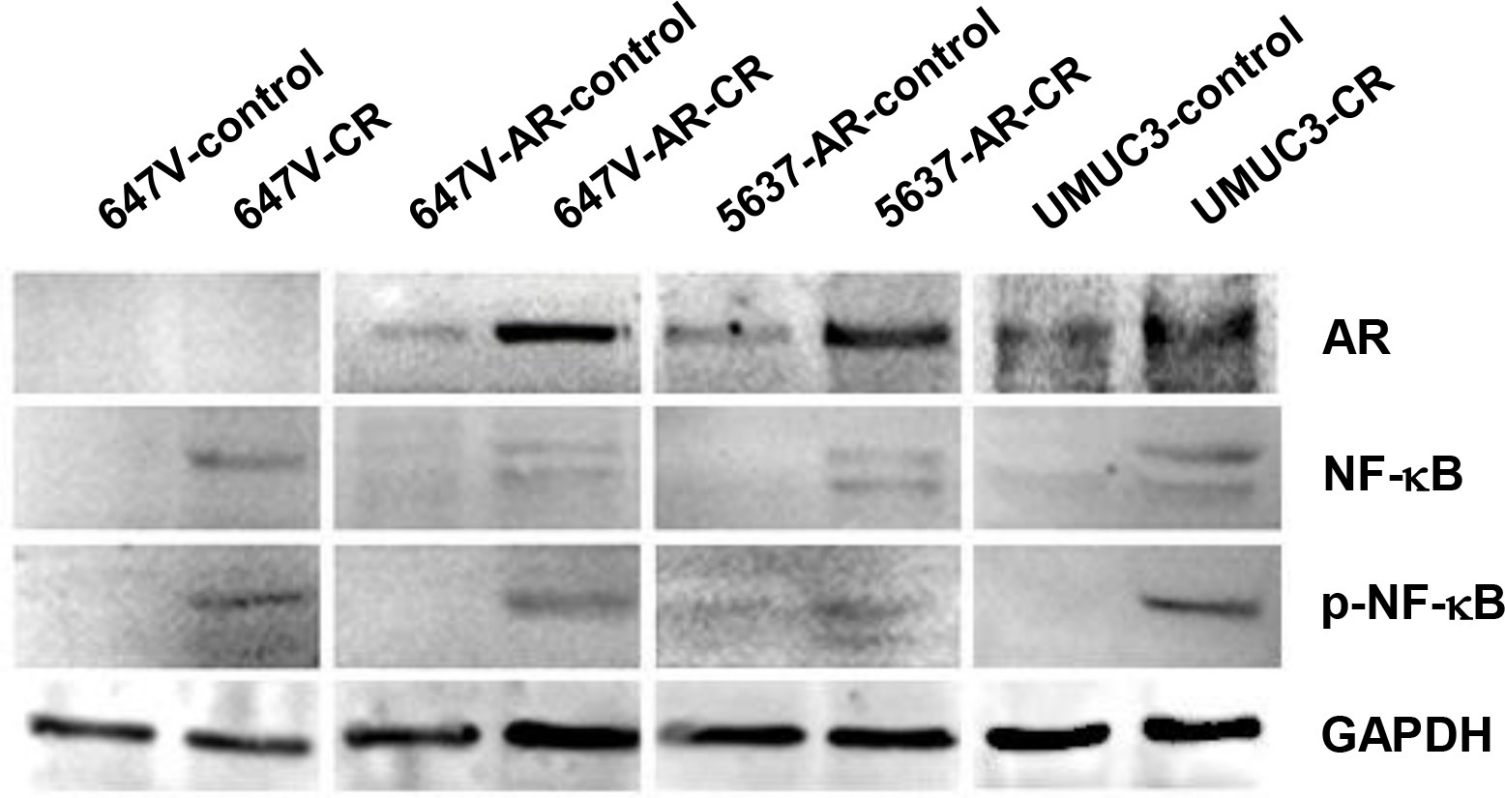
The expression of AR, NF-κB, and p-NF-κB in CR bladder cancer cells Western blotting of AR, NF-κB, and p-NF-κB in 647V-control/CR, 647V-AR-control/CR, 5637-AR-control/CR, and UMUC3-control/CR cells. Total protein extracted from each line was immunoblotted for AR (110 kDa), NF-κB (65 kDa), or p-NF-κB (65 kDa). GAPDH (37 kDa) served as an internal control.

We next compared the expression levels of NF-κB and p-NF-κB as well as AR between control and CR cells. In accordance with previous findings [28], the expression of NF-κB/p-NF-κB was considerably elevated in CR lines, compared with respective control lines (Figure 7). Additionally, AR levels were found to be higher in 647V-AR-CR, 5637-AR-CR, and UMUC3-CR cells than in control sublines 647V-AR, 5637-AR, and UMUC3, respectively. These results suggest the involvement of AR in CDDP resistance in bladder cancer cells.

### Correlations of AR/NF-κB/p-NF-κB expression with chemoresistance in bladder cancer patients

**Figure 8:**
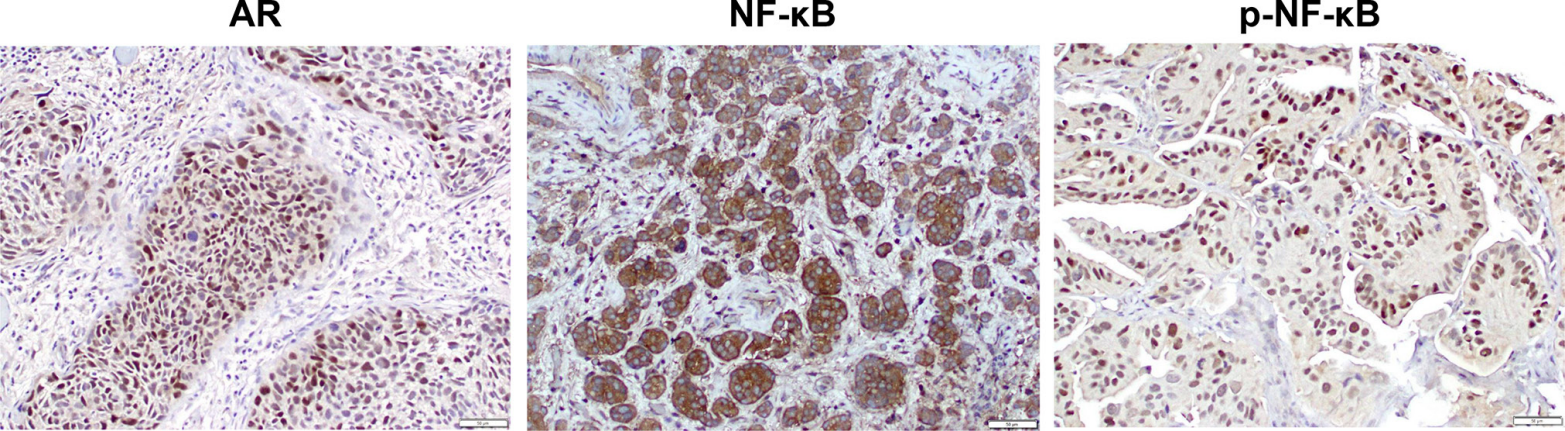
The expression of AR, NF-κB, and p-NF-κB in human bladder cancer specimens Immunohistochemistry of AR/p-NF-κB and NF-κB in bladder TMAs for which tissue specimens were collected prior to chemotherapy shows strong signals predominantly in the nucleus and cytoplasm, respectively, of tumor cells.

Finally, we immunohistochemically stained for AR, NF-κB, and p-NF-κB in our tissue microarrays (TMAs) consisting of muscle-invasive bladder cancer specimens from patients who subsequently received neoadjuvant GC therapy (Figure 8). We then compared their expression levels between responders versus non-responders to chemotherapy (Table 1). Overall, AR was positive in 19 (35%) of 55 cases, including 5 (21%) of 24 responders and 14 (45%) of 31 non-responders. Thus, AR positivity tended to correlate with resistance to chemotherapy (*P* = 0.087). These patients with AR-positive tumor included 22% of male responders *vs.* 46% of male non-responders and 20% of female responders *vs.* 40% of female non-responders. More strikingly, there was a strong correlation between p-NF-κB positivity and chemotherapy resistance (54% of responders *vs.* 81% of non-responders; *P* = 0.044). However, there was no statistically significant difference in NF-κB levels between responders and non-responders. In addition, the expression of AR and p-NF-κB in these 55 tumors was marginally correlated (*P* = 0.078), while strong (3+) positivity of NF-κB was significantly associated with p-NF-κB expression (*P* = 0.002).

**Table 1:** The expression of AR, NF-κB, and p-NF-κB in bladder cancer and response to chemotherapy

	*n*	AR expression				*P*	NF-κB expression				*P*	p-NF-κB expression				*P*
		0 (%)	1+ (%)	2+ (%)	3+ (%)		0 (%)	1+ (%)	2+ (%)	3+ (%)		0 (%)	1+ (%)	2+ (%)	3+ (%)	
All patients	55															
Responders	24	19 (79)	3 (13)	0 (0)	2 (8)	0.087^a^	0 (0)	7 (29)	11 (46)	6 (25)	0.525^b^ 0.162^c^	11 (46)	7 (29)	5 (21)	1 (4)	0.044^a^
Non-Responders	31	17 (55)	13 (42)	1 (3)	0 (0)		0 (0)	6 (19)	11(35)	14 (45)		6 (19)	15 (48)	8 (26)	2 (6)	
Male patients	45															
Responders	19	15 (78)	2 (11)	0 (0)	2 (11)	0.118^a^	0 (0)	5 (26)	9 (47)	5 (26)	0.720^b^ 0.222^c^	8 (42)	6 (32)	4 (21)	1 (5)	0.111^a^
Non-Responders	26	14 (54)	11 (42)	1 (4)	0 (0)		0 (0)	5 (19)	9 (35)	12 (46)		5 (19)	13 (50)	6 (23)	2 (8)	
Female patients	10															
Responders	5	4 (80)	1 (20)	0 (0)	0 (0)	1.000^a^	0 (0)	2 (40)	2 (40)	1 (20)	1.000^b^ 1.000^c^	3 (60)	1 (20)	1 (20)	0 (0)	0.524^a^
Non-Responders	5	3 (60)	2 (40)	0 (0)	0 (0)		0 (0)	1 (20)	2 (40)	2 (40)		1 (20)	2 (40)	2 (40)	0 (0)	

a 0 *vs.* 1+/2+/3+

b 0/1+ *vs.* 2+/3+

c 0/1+/2+ *vs.* 3+

## DISCUSSION

CDDP has been widely used for the treatment of solid malignancies, including bladder cancer. Meanwhile, underlying reasons for CDDP resistance have been extensively explored, using a variety of models of CDDP-sensitive *vs.* CDDP-resistant cancer cells, leading to identification of multifactorial mechanisms, including drug transport, accumulation, detoxification, DNA repair, transcription, and apoptosis [29–31]. Thus, molecular mechanisms responsible for CDDP resistance appear to be complicated. In the current study, we provide preclinical evidence indicating that AR activation solely results in induction of CDDP resistance in bladder cancer cells. These findings further suggest not only that AR expression may predict response to CDDP treatment but also that anti-androgenic drugs may function as sensitizers.

Preclinical findings have suggested that ADT inhibits the growth of AR-positive bladder cancer [5–10, 13, 14, 16–18]. A previous study also demonstrated that addition of dihydrotestosterone in androgen-depleted culture medium resulted in reduction of the cytotoxic effect of doxorubicin on AR-positive bladder cancer cell proliferation [23]. Similarly, in a renal pelvic urothelial carcinoma line cultured with doxorubicin or CDDP, enforced expression of AR was shown to increase cell viability, whereas CDDP cytotoxicity was not directly compared between these AR-positive versus AR-negative cells [32]. We further found that AR inactivation could increase sensitivity to CDDP in bladder cancer cells. Thus, the roles of ADT in the treatment of bladder cancer, especially in male patients, may be two-fold – direct inhibition of tumor outgrowth and enhancement of chemosensitivity. Accordingly, an additive or synergistic effect of ADT and CDDP-based chemotherapy is expected.

It has been shown that the levels of AR expression are significantly lower in bladder tumors than in non-neoplastic urothelial tissues as well as in high-grade/muscle-invasive bladder carcinomas than in low-grade/superficial tumors [21, 22]. Nonetheless, AR positivity in bladder tumors, especially in muscle-invasive carcinomas, has been associated with worse patient outcomes [8, 21]. On the other hand, none of previous studies have revealed a significant difference in the levels of AR expression between male versus female bladder tumors [22], while it remains unclear whether low levels of androgens significantly activate AR in, for instance, female tumors. In bladder cancer cell lines resistant to doxorubicin, AR expression was shown to be elevated [23]. We additionally found considerable increases in AR expression in CR lines, compared with respective control lines. Furthermore, in transurethral resection specimens from patients who underwent CDDP-based neoadjuvant chemotherapy prior to cystectomy, we detected a trend toward significance between AR expression and chemoresistance. Specifically, 22% of male responders versus 46% of male non-responders had AR-positive tumors. Thus, the status of AR expression, together with the levels of serum androgens, may serve as a predictor of chemosensitivity. In our staining, however, the degree of AR expression did not appear to have an influence on likelihood of chemoresistance, implying that the presence of a functional AR in tumor cells is critical and may suffice for inducing drug resistance. Further studies, including larger patient cohort and those who receive CDDP therapy in adjuvant settings, are needed to validate the current results. Interestingly, AR signals have also been shown to induce radioresistance in prostate cancer cells by regulating DNA repair genes that are also known to contribute to CDDP resistance [33] and the expression of ABCG2, a membrane transporter protein associated with multidrug resistance, in upper urinary tract urothelial carcinoma cells [32].

NF-κB that normally resides in the cytoplasm as an inactivated form translocates to the nucleus and thereby activates its target genes involving cell proliferation/apoptosis, angiogenesis, and cell invasion. In prostate cancer [34, 35] and endothelial [36] cells, androgens have been shown to activate NF-κB. Signaling crosstalk between NF-κB and AR has also been suggested [34, 36–38]. In particular, the p65/p50 subunit of NF-κB, as a complex with tissue transglutaminase, is able to bind to the AR promoter at NF-κB response element sites in prostate cancer cells [38]. We here showed that androgen induced the expression of NF-κB/p-NF-κB and nuclear translocation of NF-κB in bladder cancer cells.

NF-κB, as a transcription factor that induces anti-apoptotic proteins, has been considered as a key molecule for CDDP resistance [26–28]. Correspondingly, our immunohistochemistry indicated the relationship between the positivity of p-NF-κB, but not the degree of p-NF-κB or NF-κB expression, and chemoresistance. Inhibitors, including dehydroxymethylepoxyquinomicin that prevents nuclear translocation of NF-κB and its binding to DNA, have indeed been reported to not only exhibit anti-cancer activity but also confer sensitization to CDDP in CR cells [39]. However, the precise molecular mechanisms of how NF-κB signals regulate CDDP sensitivity remain far from fully understood, although NF-κB is known to regulate tumor cell sensitivity to drug-induced apoptosis. Potentially key mechanisms include activation of the anti-apoptotic bcl-2 family [40], MEKK1-mediated activation of c-JUN [41], up-regulation of anti-apoptotic c-FLIP [28], and histone modifications [42]. As aforementioned, multifactorial pathways are likely to involve CDDP resistance [29–31], and, in some of these, NF-κB may not necessarily be a central player. For instance, we have demonstrated activation of ERBB2 [8] and ELK1 [16], both of which have also been shown to induce CDDP resistance [24, 30, 31], by androgens in AR-positive bladder cancer cells.

In contrast to our current findings, in a study where the effects of androgen treatment and AR silencing on cytotoxicity of doxorubicin, 5-fluorouracil, and CDDP in bladder cancer cells were examined, androgen failed to modulate sensitivity to CDDP [23]. Differences between the previous [23] versus current studies included treatment of 1 nM dihydrotestosterone for 48 hours versus that of 10 nM R1881 for 72 hours as well as transient expression of AR-small interfering RNA versus stable expression of AR-shRNA, while UMUC3 cells were used in both studies. These differences might be relevant to the inconsistent results. Additionally, in the previous study [23], androgen did not reduce the cytotoxic activity of 5-fluorouracil. Using the same models for bladder cancer for assessing cytotoxicity of CDDP, we investigated the role of AR signals in sensitivity of gemcitabine and found no significant effects of AR overexpression or silencing (data not shown). Thus, NF-κB appears to play an insignificant role in AR-induced gemcitabine resistance, if any, in bladder cancer cells. Nonetheless, NF-κB activation was shown to be a basis for gemcitabine resistance in pancreatic cancer cells [43].

In conclusion, we demonstrate preclinical evidence indicating that AR activation contributes to CDDP resistance in bladder cancer cells presumably via NF-κB activation. Therefore, patients with AR-negative and p-NF-κB-negative tumor are more likely to respond to CDDP-based chemotherapy. In contrast, those with AR-positive or p-NF-κB-positive tumor may be resistant to CDDP therapy and are anticipated to benefit from combined anti-AR therapy. Thus, depending on the gender and hormone milieu of the patients as well as AR expression profile in the tumors, comedication with ADT may be useful for overcoming chemoresistance. Further investigations are required to determine the effects of anti-AR therapy on chemosensitivity, especially in animal models for bladder cancer, and to elucidate the precise mechanisms of how AR signals involve CDDP resistance in bladder cancer cells.

## MATERIALS AND METHODS

### Antibodies and chemicals

Anti-AR (N-20), anti-NF-κB (sc-109), and anti-GAPDH (6c5) antibodies and an anti-p-NF-κB antibody (Ser536) were purchased from Santa Cruz Biotechnology and Cell Signaling Technology, respectively. We obtained methyltrienolone (R1881) from PerkinElmer, and HF and CDDP from Sigma-Aldrich.

### Cell lines

Human urothelial carcinoma cell lines, UMUC3 and 5637, were originally obtained from the American Type Culture Collection (Manassas, VA). Another human urothelial carcinoma cell line, 647V, was used in our previous studies [5, 15–18, 24, 44]. All these lines were recently authenticated, using GenePrint 10 System (Promega), by the institutional core facility.

Cell lines stably expressing a full-length wild-type human AR (5637-AR and 647V-AR) or vector only (5637-V and 647V-V) were established, using a lentivirus vector (pWPI-AR or pWPI-control) with psPAX2 envelope and pMD2.G packaging plasmids, as we described previously [8, 15]. Similarly, stable AR knockdown/control cell lines [UMUC3-AR-short hairpin RNA (shRNA)/UMUC3-control-shRNA] were established, using a retrovirus vector pMSCV/U6-AR-shRNA or pMSCV/U6-control-shRNA [8].

CDDP-resistant cell lines, 647V-CR, 647V-AR-CR, 5637-AR-CR, and UMUC3-CR, were then established from 647V, 647V-AR, 5637-AR, and UMUC3 cells, respectively, by stepwise, continuous treatment with CDDP (*e.g.* 0.2–2.0 μM) for at least 12-weeks.

All these cell lines were maintained in Dulbecco's modified Eagle's medium (Mediatech) supplemented with 10% FBS at 37°C in a humidified atmosphere of 5% CO2. Cells were then cultured in phenol red-free medium supplemented with either 10% FBS or 10% CS-FBS at least 24 hours before experimental treatment.

### Western blot

Equal amounts of proteins obtained from cell extracts were subjected to sodium dodecyl sulfate-polyacrylamide gel electrophoresis, transferred to polyvinylidene difluoride membranes electronically, blocked, and incubated with a specific primary antibody. The membrane was then incubated with a secondary antibody, and specific signals were detected, using a LI-COR imaging system.

### MTT assay

We used the MTT (thiazolyl blue) assay to assess cell viability, as described previously [15], with minor modifications. Briefly, cells (2–6 × 10^3^) seeded in 96-well plates were cultured for 72 h in the presence or absence of CDDP and then incubated with 0.5 mg/mL of MTT (Sigma) in 100 μL of medium for 3 h at 37°C. MTT was solved by DMSO, and the absorbance was measured at a wavelength of 570 nm with background subtraction at 630 nm.

### RT and real-time PCR

Total RNA isolated from cultured cells by TRIzol (Invitrogen, Carlsbad, CA) was reverse transcribed, using 1 μmol/l oligo(dT) primers and four units of Ominiscript reverse transcriptase (Qiagen). Real-time PCR was then performed, using RT2 SYBR Green FAST Mastermix (Qiagen), as described previously [16]. The primer sequences are: 5′-AACAGAGAGGATTTCGTTTCC-3′ (forward) and 5′-TTTGACCTGAGGGTAAGACTTCT-3′ (reverse) for NF-κB; and 5′-TGTGGGCATCAATGGATTTGG-3′ (forward) and 5′-ACACCATGTATTCCGGGTCAAT −3′(reverse) for GAPDH.

### Immunofluorescent staining

Immunofluorescent staining was performed, as described previously [13, 16, 44], with minor modifications. Briefly, cells plated onto chamber slides (8-well Nunc Lab-Tek, Thermo Scientific) were cultured in medium containing ethanol or R1881 for 24 h, and the adherent cells were fixed with 4% paraformaldehyde. After being blocked, a primary antibody (NF-κB) was incubated at 4°C overnight, and Alexa 488 conjugated secondary antibody (diluted 1:500, Invitrogen) was added for 1 h at 37°C. Fluorescence images were acquired with an Olympus FV1000 confocal microscope and nuclear expression of the proteins was quantified by a single observer who was unaware of the treatment group for the cells.

### Immunohistochemistry

We retrieved bladder tissue specimens obtained by transurethral resection for TMA construction. Appropriate approval from the institutional review board was obtained before construction and use of the TMAs. These bladder TMAs constructed previously [21, 24, 45] included cases of high-grade muscle-invasive urothelial carcinoma that received GC neoadjuvant chemotherapy prior to radical cystectomy. Patients who received only 3 cycles of GC with dose reduction or ≤ 2 cycles of GC were excluded from the analysis. Responders (*n* = 24) and non-responders (*n* = 31) to the neoadjuvant therapy were pathologically defined as the absence (≤ pT1N0M0) and presence (≥ pT2, pN1-3, and/or M1), respectively, of muscle-invasive, extravesical, or metastatic disease at the time of cystectomy [46].

Immunohistochemistry was performed on the sections (5 μm thick) from the bladder TMAs, using a primary antibody to AR (dilution 1:200), NF-κB (dilution 1: 200), or p-NF-κB (dilution 1: 50) and a broad spectrum secondary antibody (Invitrogen), as described previously [8, 21]. All stains were manually quantified by a single pathologist (H.M.) blinded to sample identify. The German immunoreactive scores calculated by multiplying the percentage of immunoreactive cells (0% = 0; 1–10% = 1; 11–50% = 2; 51–80% = 3; 81–100% = 4) by staining intensity (negative = 0; weak = 1; moderate = 2; strong = 3) were considered negative (0; 0–1), weakly positive (1+; 2–4), moderately positive (2+; 6–8), and strongly positive (3+; 9–12).

### Statistical analysis

Student's *t*-test was used to analyze differences in variables with a continuous distribution. The Fisher exact test or chi-square test was used to evaluate the associations between categorized variables. *P* values less than 0.05 was considered statistically significant.
